# Physical activity and fat-free mass during growth and in later life

**DOI:** 10.1093/ajcn/nqab260

**Published:** 2021-09-03

**Authors:** Klaas R Westerterp, Yosuke Yamada, Hiroyuki Sagayama, Philip N Ainslie, Lene F Andersen, Liam J Anderson, Lenore Arab, Issaad Baddou, Kweku Bedu-Addo, Ellen E Blaak, Stephane Blanc, Alberto G Bonomi, Carlijn V C Bouten, Pascal Bovet, Maciej S Buchowski, Nancy F Butte, Stefan G J A Camps, Graeme L Close, Jamie A Cooper, Sai K Das, Richard Cooper, Lara R Dugas, Ulf Ekelund, Sonja Entringer, Terrence Forrester, Barry W Fudge, Annelies H Goris, Michael Gurven, Catherine Hambly, Asmaa El Hamdouchi, Marije B Hoos, Sumei Hu, Noorjehan Joonas, Annemiek M Joosen, Peter Katzmarzyk, Kitty P Kempen, Misaka Kimura, William E Kraus, Robert F Kushner, Estelle V Lambert, William R Leonard, Nader Lessan, Corby K Martin, Anine C Medin, Erwin P Meijer, James C Morehen, James P Morton, Marian L Neuhouser, Theresa A Nicklas, Robert M Ojiambo, Kirsi H Pietiläinen, Yannis P Pitsiladis, Jacob Plange-Rhule, Guy Plasqui, Ross L Prentice, Roberto A Rabinovich, Susan B Racette, David A Raichlen, Eric Ravussin, Rebecca M Reynolds, Susan B Roberts, Albertine J Schuit, Anders M Sjödin, Eric Stice, Samuel S Urlacher, Giulio Valenti, Ludo M Van Etten, Edgar A Van Mil, Jonathan C K Wells, George Wilson, Brian M Wood, Jack Yanovski, Tsukasa Yoshida, Xueying Zhang, Alexia J Murphy-Alford, Cornelia U Loechl, Amy H Luke, Herman Pontzer, Jennifer Rood, Dale A Schoeller, William W Wong, John R Speakman, International Atomic Energy Agency Doubly Labeled Water database group, Stefan Branth, Lisa H Colbert, Niels C De Bruin, Alice E Dutman, Sölve Elmståhl, Mikael Fogelholm, Tamara Harris, Rik Heijligenberg, Hans U Jorgensen, Christel L Larsson, Elisabet M Rothenberg, Margaret McCloskey, Gerwin A Meijer, Daphne L Pannemans, Sabine Schulz, Rita Van den Berg-Emons, Wim G Van Gemert, W Wilhelmine, Venne Verboeket-van de, Jeanine A Verbunt, Renaat M Philippaerts, Amy Subar, Minna Tanskanen, Ricardo Uauy, Erica J Velthuis-te Wierik

**Affiliations:** School of Nutrition and Translational Research in Metabolism, University of Maastricht, Maastricht, The Netherlands; National Institute of Health and Nutrition, National Institutes of Biomedical Innovation, Health and Nutrition, Tokyo, Japan; Institute for Active Health, Kyoto University of Advanced Science, Kyoto, Japan; Faculty of Health and Sport Sciences, University of Tsukuba, Tsukuba, Japan; Research Institute for Sport and Exercise Sciences, Liverpool John Moores University, Liverpool, United Kingdom; Department of Nutrition, Institute of Basic Medical Sciences, University of Oslo, Oslo, Norway; Research Institute for Sport and Exercise Sciences, Liverpool John Moores University, Liverpool, United Kingdom; Crewe Alexandra Football Club, Crewe, United Kingdom; David Geffen School of Medicine, University of California, Los Angeles, CA, USA; Unité Mixte de Recherche en Nutrition et Alimentation, CNESTEN–Université Ibn Tofail URAC39, Regional Designated Center of Nutrition Associated with African Regional Agreement for Research/International Atomic Energy Agency, Rabat, Morocco; Department of Physiology, Kwame Nkrumah University of Science and Technology, Kumasi, Ghana; Faculty of Health, Medicine and Life Sciences, Maastricht University, Maastricht, Netherlands; Nutritional Sciences, University of Wisconsin, Madison, WI, USA; Institut Pluridisciplinaire Hubert Curien. CNRS Université de Strasbourg, UMR7178, Strasbourg, France; Phillips Research, Eindhoven, The Netherlands; Department of Biomedical Engineering and Institute for Complex Molecular Systems, Eindhoven University of Technology, Eindhoven, The Netherlands; University Center for Primary Care and Public Health (Unisanté), Lausanne, Switzerland; Division of Gastroenterology, Hepatology and Nutrition, Department of Medicine, Vanderbilt University, Nashville, TN, USA; Department of Pediatrics, Baylor College of Medicine, USDA/Agricultural Research Service Children's Nutrition Research Center, Houston, TX, USA; Faculty of Health, Medicine and Life Sciences, Maastricht University, Maastricht, Netherlands; Research Institute for Sport and Exercise Sciences, Liverpool John Moores University, Liverpool, United Kingdom; Nutritional Sciences, University of Wisconsin, Madison, WI, USA; Jean Mayer USDA Human Nutrition Research Center on Aging at Tufts University, Boston, MA, USA; Department of Public Health Sciences, Parkinson School of Health Sciences and Public Health, Loyola University, Maywood, IL, USA; Department of Public Health Sciences, Parkinson School of Health Sciences and Public Health, Loyola University, Maywood, IL, USA; Department of Sport Medicine, Norwegian School of Sport Sciences, Oslo, Norway; Institute of Medical Psychology, Charité—Universitätsmedizin Berlin, corporate member of Freie Universität Berlin, Humboldt-Universität zu Berlin, and Berlin Institute of Health (BIH), Berlin, Germany; Department of Pediatrics, University of California Irvine, Irvine, CA, USA; Solutions for Developing Countries, University of the West Indies, Mona, Kingston, Jamaica; Institute of Biomedical and Life Sciences, University of Glasgow, Glasgow, United Kingdom; Faculty of Health, Medicine and Life Sciences, Maastricht University, Maastricht, Netherlands; Department of Anthropology, University of California Santa Barbara, Santa Barbara, CA, USA; Institute of Biological and Environmental Sciences, University of Aberdeen, Aberdeen, United Kingdom; Unité Mixte de Recherche en Nutrition et Alimentation, CNESTEN–Université Ibn Tofail URAC39, Regional Designated Center of Nutrition Associated with African Regional Agreement for Research/International Atomic Energy Agency, Rabat, Morocco; Faculty of Health, Medicine and Life Sciences, Maastricht University, Maastricht, Netherlands; State Key Laboratory of Molecular Developmental Biology, Institute of Genetics and Developmental Biology, Chinese Academy of Sciences, Beijing, China; Central Health Laboratory, Ministry of Health and Wellness, Port Louis, Mauritius; Faculty of Health, Medicine and Life Sciences, Maastricht University, Maastricht, Netherlands; Pennington Biomedical Research Center, Baton Rouge, LA, USA; Faculty of Health, Medicine and Life Sciences, Maastricht University, Maastricht, Netherlands; National Institute of Health and Nutrition, National Institutes of Biomedical Innovation, Health and Nutrition, Tokyo, Japan; Department of Medicine, Duke University, Durham, NC, USA; Department of Medicine, Northwestern University, Chicago, IL, USA; Research Unit for Exercise Science and Sports Medicine, University of Cape Town, Cape Town, South Africa; Department of Anthropology, Northwestern University, Evanston, IL, USA; Imperial College London Diabetes Centre, Imperial College London, London, United Kingdom; Pennington Biomedical Research Center, Baton Rouge, LA, USA; Department of Nutrition, Institute of Basic Medical Sciences, University of Oslo, Oslo, Norway; Department of Nutrition and Public Health, Faculty of Health and Sport Sciences, University of Agder, Kristiansand, Norway; Faculty of Health, Medicine and Life Sciences, Maastricht University, Maastricht, Netherlands; Research Institute for Sport and Exercise Sciences, Liverpool John Moores University, Liverpool, United Kingdom; The FA Group, Burton-Upon-Trent, United Kingdom; Research Institute for Sport and Exercise Sciences, Liverpool John Moores University, Liverpool, United Kingdom; Division of Public Health Sciences, Fred Hutchinson Cancer Research Center and School of Public Health, University of Washington, Seattle, WA, USA; Department of Pediatrics, Baylor College of Medicine, USDA/Agricultural Research Service Children's Nutrition Research Center, Houston, TX, USA; Department of Medical Physiology, Moi University, Eldoret, Kenya; Department of Biomedical Sciences, University of Global Health Equity, Butaro, Rwanda; Helsinki University Central Hospital, Helsinki, Finland; Collaborating Centre of Sports Medicine, University of Brighton, Eastbourne, United Kingdom; Department of Physiology, Kwame Nkrumah University of Science and Technology, Kumasi, Ghana; Department of Nutrition and Movement Sciences, Maastricht University, Maastricht, The Netherlands; Division of Public Health Sciences, Fred Hutchinson Cancer Research Center and School of Public Health, University of Washington, Seattle, WA, USA; Department of Respiratory Medicine, University of Edinburgh, Edinburgh, United Kingdom; Program in Physical Therapy and Department of Medicine, Washington University School of Medicine, St Louis, MO, USA; Biological Sciences and Anthropology, University of Southern California, Los Angeles, CA, USA; Pennington Biomedical Research Center, Baton Rouge, LA, USA; Centre for Cardiovascular Sciences, Queen's Medical Research Institute, University of Edinburgh, Edinburgh, United Kingdom; Jean Mayer USDA Human Nutrition Research Center on Aging at Tufts University, Boston, MA, USA; School of Social and Behavioural Sciences, University of Tilburg, Tilburg, The Netherlands; Department of Nutrition, Exercise and Sports, Copenhagen University, Copenhagen, Denmark; Department of Psychiatry, Stanford University, Stanford, CA, USA; Department of Anthropology, Baylor University, Waco, TX, USA; Faculty of Health, Medicine and Life Sciences, Maastricht University, Maastricht, Netherlands; Faculty of Health, Medicine and Life Sciences, Maastricht University, Maastricht, Netherlands; Faculty of Health, Medicine and Life Sciences, and Faculty of Science and Engineering, Maastricht University, Maastricht, The Netherlands; Population, Policy and Practice Research and Teaching Department, UCL Great Ormond Street Institute of Child Health, London, United Kingdom; Research Institute for Sport and Exercise Sciences, Liverpool John Moores University, Liverpool, United Kingdom; Department of Antropology, University of California Los Angeles, Los Angeles, CA, USA; Department of Human Behavior, Ecology, and Culture, Max Planck Institute for Evolutionary Anthropology, Leipzig, Germany; Section on Growth and Obesity, Division of Intramural Research, Eunice Kennedy Shriver National Institute of Child Health and Human Development, NIH, Bethesda, MD, USA; Faculty of Health and Sport Sciences, University of Tsukuba, Tsukuba, Japan; Institute of Biological and Environmental Sciences, University of Aberdeen, Aberdeen, United Kingdom; State Key Laboratory of Molecular Developmental Biology, Institute of Genetics and Developmental Biology, Chinese Academy of Sciences, Beijing, China; Nutritional and Health-Related Environmental Studies Section, Division of Human Health, International Atomic Energy Agency, Vienna, Austria; Nutritional and Health-Related Environmental Studies Section, Division of Human Health, International Atomic Energy Agency, Vienna, Austria; Division of Epidemiology, Department of Public Health Sciences, Loyola University School of Medicine, Maywood, IL, USA; Evolutionary Anthropology, Duke University, Durham, NC, USA; Duke Global Health Institute, Duke University, Durham, NC, USA; Pennington Biomedical Research Center, Baton Rouge, LA, USA; Biotech Center and Nutritional Sciences, University of Wisconsin, Madison, WI, USA; Department of Pediatrics, Baylor College of Medicine, USDA/Agricultural Research Service Children's Nutrition Research Center, Houston, TX, USA; Institute of Biological and Environmental Sciences, University of Aberdeen, Aberdeen, United Kingdom; State Key Laboratory of Molecular Developmental Biology, Institute of Genetics and Developmental Biology, Chinese Academy of Sciences, Beijing, China; Center for Energy Metabolism and Reproduction, Shenzhen Institutes of Advanced Technology, Chinese Academy of Sciences, Shenzhen, China; CAS Center of Excellence in Animal Evolution and Genetics, Kunming, China; University of Uppsala, Uppsala, Sweden; Kinesiology, University of Wisconsin, Madison, WI, USA; Erasmus University, Rotterdam, Netherlands; TNO Quality of Life, Zeist, Netherlands; Lund University, Lund, Sweden; Department of Food and Nutrition, Helsinki, Finland; NIH, Bethesda, MD, USA; Academic Medical Center of Amsterdam University, Amsterdam, Netherlands; Bispebjerg Hospital, Copenhagen, Denmark; University of Gothenburg, Gothenburg, Sweden; Royal Belfast Hospital for Sick Children, Belfast, United Kingdom; Maastricht University, Maastricht, Netherlands; Katholieke University Leuven, Leuven, Belgium; Epidemiology and Genomics, Division of Cancer Control, NIH, Bethesda, MD, USA; University of Jyväskilä, Jyväskilä, Finland; Institute of Nutrition and Food Technology (INTA), University of Chile, Santiago, Chile; TNO Nutrition and Food Research Institute, Zeist, Netherlands

**Keywords:** physical activity level, age, energy expenditure, body composition, doubly labeled water

## Abstract

**Background:**

Physical activity may be a way to increase and maintain fat-free mass (FFM) in later life, similar to the prevention of fractures by increasing peak bone mass.

**Objectives:**

A study is presented of the association between FFM and physical activity in relation to age.

**Methods:**

In a cross-sectional study, FFM was analyzed in relation to physical activity in a large participant group as compiled in the International Atomic Energy Agency Doubly Labeled Water database. The database included 2000 participants, age 3–96 y, with measurements of total energy expenditure (TEE) and resting energy expenditure (REE) to allow calculation of physical activity level (PAL = TEE/REE), and calculation of FFM from isotope dilution.

**Results:**

PAL was a main determinant of body composition at all ages. Models with age, fat mass (FM), and PAL explained 76% and 85% of the variation in FFM in females and males < 18 y old, and 32% and 47% of the variation in FFM in females and males ≥ 18 y old, respectively. In participants < 18 y old, mean FM-adjusted FFM was 1.7 kg (95% CI: 0.1, 3.2 kg) and 3.4 kg (95% CI: 1.0, 5.6 kg) higher in a very active participant with PAL = 2.0 than in a sedentary participant with PAL = 1.5, for females and males, respectively. At age 18 y, height and FM–adjusted FFM was 3.6 kg (95% CI: 2.8, 4.4 kg) and 4.4 kg (95% CI: 3.2, 5.7 kg) higher, and at age 80 y 0.7 kg (95% CI: −0.2, 1.7 kg) and 1.0 kg (95% CI: −0.1, 2.1 kg) higher, in a participant with PAL = 2.0 than in a participant with PAL = 1.5, for females and males, respectively.

**Conclusions:**

If these associations are causal, they suggest physical activity is a major determinant of body composition as reflected in peak FFM, and that a physically active lifestyle can only partly protect against loss of FFM in aging adults.

See corresponding editorial on page 1579.

## Introduction

Physical activity provides a variety of health benefits. Physically active individuals sleep better and function better (1). In addition, physical activity can be an effective lifestyle behavior to maximize fat-free mass (FFM), as a proxy for muscle mass, during growth. Youth physical activity is positively associated with bone mass accrual and bone structure (2, 3). Physical activity may be a way to increase and maintain FFM to prevent sarcopenia in later life, similar to the prevention of fractures by increasing peak bone mass (4–6).

Skeletal muscle accounts for about half of FFM. Muscle mass and bone mass are closely related throughout life, and FFM is the strongest determinant of whole-body bone mineral content. Modeling and remodeling processes that regulate bone strength potentially explain these relations, depending on the forces acting on the bones (7, 8). Physical activity positively affects FFM accretion from birth onwards (9). Physical activity during adolescence has been associated with greater FFM in both sexes (10). Habitual physical activity has been shown to have a significant independent effect on the growth of FFM during adolescence (11). These results support recommendations for sustained physical activity participation during the growing years (12).

FFM peaks in early adulthood (13). A cross-sectional analysis of a large multiethnic sample, ranging in age from 18 to 110 y, resulted in a quadratic model for FFM in relation to age with a peak FFM at similar ages for Caucasians, African Americans, Hispanics, and Asians. The estimated turning point, where growth ended and FFM started to decline, was in the mid-40s for females and mid-20s for males (13). Physical activity is likely to have a role in preventing FFM loss at later ages. A cross-sectional study showed that higher physical activity was associated with higher FFM in participants aged 60–64 y (14). A longitudinal study in participants aged 65–84 y showed that greater physical activity retained a greater FFM over 5 y of observation (15). On the other hand, a cross-sectional study in 529 participants aged 18–96 y suggested that greater physical activity was not associated with higher FFM (16). Two longitudinal studies, the first in 904 participants aged 67–84 y and the second in 302 participants aged 70–82 y, also showed that changes in FFM over 5 y were not associated with physical activity level (PAL), when controlled for potential confounding variables (17, 18). Thus, there is still controversy on the relation between physical activity and FFM at later ages.

Here, the focus is on physical activity and FFM accrual during early and later life. A cross-sectional analysis was performed in a large participant group, deriving physical activity from doubly labeled water–measured energy expenditure. Thus, physical activity was quantified with a criterion measure (19).

## Methods

The analysis included daily total energy expenditure (TEE) measurements as compiled in the International Atomic Energy Agency Doubly Labeled Water database (established to pool doubly labeled water data across multiple studies), version 3.1.2 (20). All data were recalculated with the same standard methodology for human doubly labeled water studies as published recently (21). The analysis was restricted to TEE measures accompanied by measurements of resting energy expenditure (REE), to allow calculation of PAL (TEE/REE). REE was measured under postabsorptive, thermoneutral, and resting conditions with a ventilated hood, or during an early morning resting interval, directly after waking up and before having breakfast, in a respiration chamber.

**FIGURE 1:**
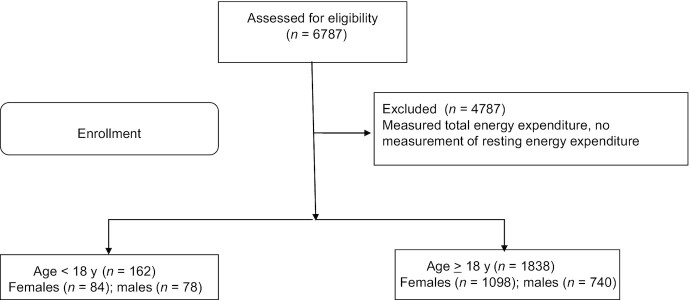
Participant flowchart.

The database included 2000 participants (1182 females and 818 males) with measurements on TEE and REE to allow calculation of PAL (**Figure 1**). The age range of the participants was 3–96 y. The data analysis did not include participants with muscle wasting or participants with diseases affecting REE. All TEE measurements were performed under habitual daily life conditions, neutral energy balance, and before any study intervention. FFM was derived from total body water as measured with isotope dilution, a method directly derived from carcass analysis and thus 1 of the 2 single-indirect methods for body composition (22).

Associations between physical activity and FFM can be confounded by fat mass (FM) because gains or losses in fat typically lead to respective gains or losses in FFM (23). Changes in body weight and body composition are primarily a function of energy balance. Consequently, changes in FM and FFM are not independent (24). Energy balance–related body mass changes are generally assumed to consist of 75% as FM and 25% as FFM, which is known as the “quarter FFM” rule (25). Refinements of the quarter FFM rule were developed for specific situations like diet-induced weight change in extremely lean participants or participants with obesity (26, 27). Whatever rule applies for the relation between energy balance–induced changes in FM and FFM, FM should be included as an independent variable in an analysis on physical activity and FFM.

Data analysis was performed separately for participants < 18 y old and for participants ≥ 18 y old. For participants < 18 y old, the relation between FFM and PAL was assessed in a multiple regression model accounting for FM and age. To allow body composition comparisons between participants ≥ 18 y old, FFM and FM were expressed as indexes, the fat-free mass index (FFMI) and fat mass index (FMI), respectively, where FFMI = FFM/height^2^ and FMI = FM/height^2^ (FFM and FM in kg and height in m). In this way we corrected for differences in height, in analogy with the BMI of Quetelet: BMI = FFMI + FMI (28). Unfortunately, the index fails to adjust for height differences in participants during growth (29). Thus, data analysis was performed separately for participants < 18 y old, using unadjusted FFM and FM as measures for body composition. Models were generated separately for females and males. In participants ≥ 18 y old, 4 models were applied in a top-down procedure, with FFMI as the outcome variable:

- Model 1: age, FMI, PAL.
- Model 2: age, FMI, PAL, age^2^.
- Model 3: age, FMI, PAL, age^2^, age*PAL.
- Model 4: age, FMI, PAL, age^2^, age*PAL, age^2^*PAL.

Because the linearity assumption for age was violated, a quadratic term (age^2^) needed to be included. The model explaining most variation in FFM from age-, FM-, and PAL-differences between participants was model 3. For females, model 3 was better than model 2, and model 4 was not better than model 3. For males, model 3 was as good as model 2, and model 4 was not better than model 3. Thus, model 3 was chosen for both sexes. Model 3 was checked for (multi)collinearity after centering for age, resulting in the same model fit and in condition indexes <30 (18.2 for females, 16.6 for males), indicating there was no collinearity problem.

## Results

**FIGURE 2:**
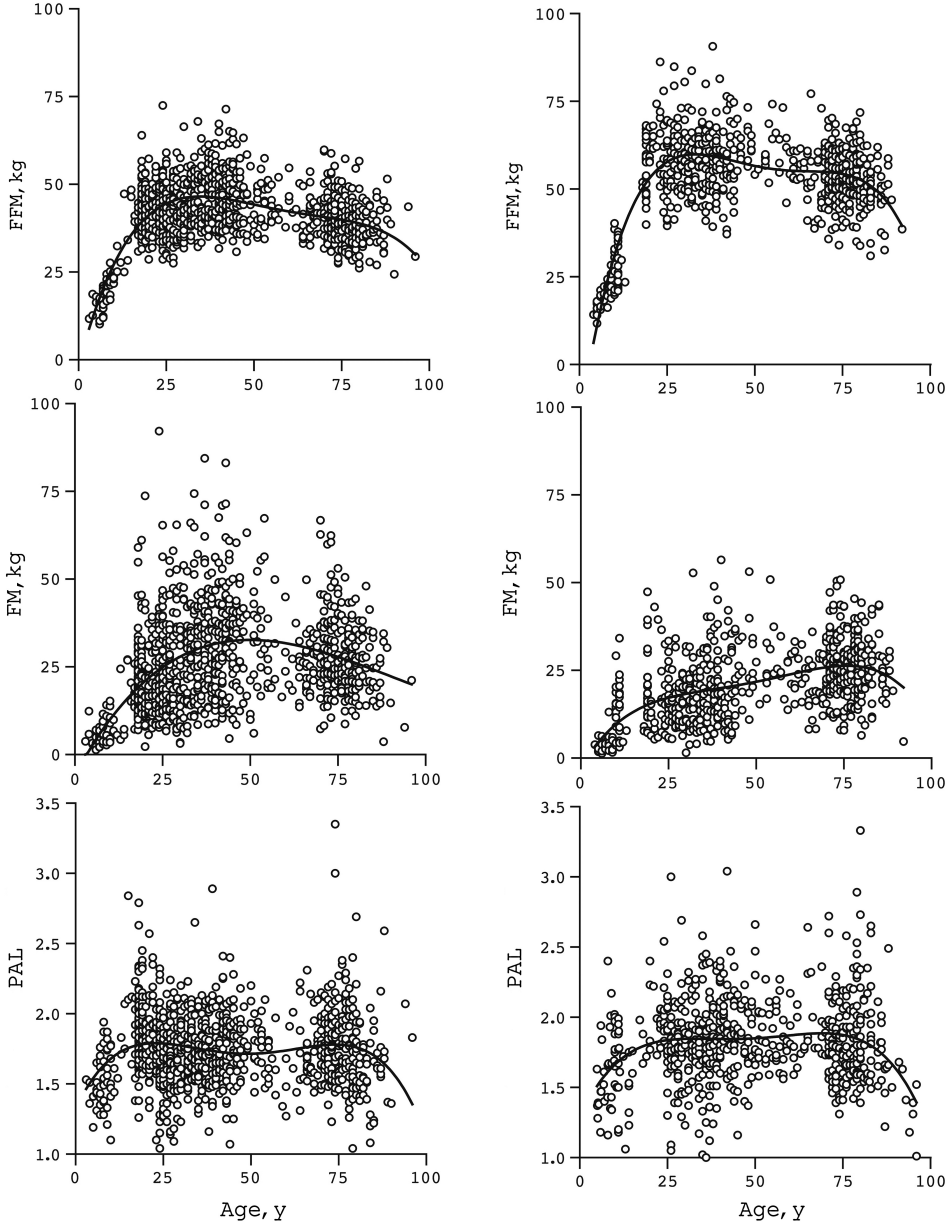
FFM, FM, and PAL, plotted as a function of age. Values for 2000 participants—1182 females (left) and 818 males (right)—with a 4th-order polynomial curve fit. FFM, fat-free mass; FM, fat mass; PAL, physical activity level.

FFM was highest, around age 30 y, in females and males (**Figure 2**). The mean of peak FFM was 47 kg in females and 60 kg in males. Females showed a higher mean FM than males already at early ages. Mean PAL was similar in females and males at all ages (**Table 1**). The typical mean PAL value was ∼1.5 in the youngest (age < 10 y) and oldest (age > 80 y) participants (Figure 2). At adult age, from 18 to 80 y, PAL values generally ranged between a minimum of 1.1 and a maximum of 2.5 with a mean ± SD value of 1.71 ± 0.26 for females and 1.78 ± 0.30 for males.

**TABLE 1:** Participant characteristics^1^

Characteristics	Females	Males
<18 y old		
*n*	84	78
Fat-free mass, kg	26.7 ± 10.9	26.9 ± 10.2
Fat mass, kg	10.8 ± 10.4	9.2 ± 7.7
PAL	1.61 ± 0.30	1.62 ± 0.29
≥18 y old		
*n*	1098	740
Fat-free mass index, kg/m^2^	16.2 ± 2.3	18.5 ± 2.2
Fat mass index, kg/m^2^	10.3 ± 4.9	7.4 ± 3.6
PAL	1.71 ± 0.26	1.78 ± 0.30

1 Values are mean ± SD unless otherwise indicated. PAL, physical activity level.

In participants < 18 y old (*n* = 162), FFM was significantly higher in individuals with an older age, higher FM, and higher PAL (**Table 2**):

- Females, FFM (kg) = −1.53 + 1.90 Age (y) + 0.21 FM (kg) + 3.34 PAL, *R*^2^ = 0.85;
- Males, FFM (kg) = −7.42 + 2.00 Age (y) + 0.39 FM (kg) + 6.90 PAL, *R*^2^ = 0.76.

**TABLE 2:** Sources of variation in FFM in participants <18 y old^1^

	Unstandardized coefficient (B)	95% CI for B	*P*
Females			
(constant)	−1.53	−6.21, 3.14	0.516
Age	1.90	1.63, 2.16	<0.001
FM	0.21	0.10, 0.31	<0.001
PAL	3.34	0.21, 6.47	0.037
Males			
(constant)	−7.42	−14.45, −0.39	0.039
Age	2.00	1.57, 2.44	<0.001
FM	0.39	0.22, 0.57	<0.001
PAL	6.90	2.66, 11.15	0.002

1 Values are coefficients and *P* values from a multiple regression model of FFM (kg) as a function of age (y), FM (kg), and PAL, in females (*n* = 84, *R*^2^ = 0.85) and males (*n* = 78, *R*^2^ = 0.76). FFM, fat-free mass; FM, fat mass; PAL, physical activity level.

Thus, mean FM-adjusted FFM was 1.7 kg (95% CI: 0.1, 3.2 kg) and 3.4 kg (95% CI: 1.0, 5.6 kg) higher in a very active participant with PAL = 2.0 than in a sedentary participant with PAL = 1.5, for females and males, respectively.

In participants ≥ 18 y old (*n* = 1838), FFMI was significantly higher in participants with a higher FMI and PAL for both sexes (**Table 3**):

- Females, FFMI (kg/m^2^) = 7.15 + 0.094 Age (y) − 0.001 Age^2^ (y) + 0.312 FMI (kg/m^2^) + 3.214 PAL − 0.033 Age*PAL (y), *R*^2^ = 0.47;
- Males, FFMI (kg/m^2^) = 9.084 + 0.141 Age (y) − 0.001 Age^2^ (y) + 0.308 FMI (kg/m^2^) + 3.557 PAL − 0.036 Age*PAL (y), *R*^2^ = 0.32.

**TABLE 3:** Sources of variation in FFMI in participants ≥ 18 y old^1^

	Unstandardized coefficient (B)	95% CI for B	*P*
Females			
(constant)	7.150	5.466, 8.838	<0.001
Age	0.094	0.049, 0.140	<0.001
FMI	0.312	0.290, 0.334	<0.001
PAL	3.214	2.379, 4.050	<0.001
Age^2^	−0.001	−0.001, 0.000	<0.001
Age*PAL	−0.033	−0.050, −0.017	<0.001
Males			
(constant)	9.084	6.674, 11.494	<0.001
Age	0.141	0.080, 0.201	<0.001
FMI	0.308	0.267, 0.349	<0.001
PAL	3.557	2.442, 4.671	<0.001
Age^2^	−0.001	−0.001, −0.000	<0.001
Age*PAL	−0.036	−0.056, −0.016	<0.001

1 Values are coefficients and *P* values from a multiple regression model of FFMI (kg/m^2^) as a function of age (y), FMI (kg/m^2^), PAL, and interactions with age (y), in females (*n* = 1098, *R*^2^ = 0.47) and males (*n* = 740, *R*^2^ = 0.32). FFMI, fat-free mass index; FMI, fat mass index; PAL, physical activity level.

At age 18 y, mean FMI-adjusted FFMI was 1.3 kg/m^2^ (95% CI: 1.0, 1.6 kg/m^2^) and 1.4 kg/m^2^ (95% CI: 1.0, 1.8 kg/m^2^) higher in a very active participant with PAL = 2.0 than in a sedentary participant with PAL = 1.5, for females and males, respectively. The differences in FFMI imply, for a typical female with height 1.65 m and male with height 1.75 m, a mean FM-adjusted FFM difference of 3.6 kg (95% CI: 2.8, 4.4 kg) and 4.4 kg (95% CI: 3.2, 5.7 kg), respectively. The positive association between FMI-adjusted FFMI and PAL was smaller the older the participant (Table 3). Thus, at age 80 y, the differences in FFM between a sedentary and very active female and male were 0.7 kg (95% CI: −0.2, 1.7 kg) and 1.0 kg (95% CI: −0.1, 2.1 kg), respectively.

Participants with a higher FM had a higher FFM. The mean of the coefficient was 0.21 and 0.39 kg FFM/kg FM, or 17% and 39% FFM/kg body mass, in females and males < 18 y old, respectively. At later ages (>18 y old), the mean of the coefficient was 0.312 and 0.308 kg FFM/kg FM in females and males, respectively, or 24% FFM/kg body mass.

## Discussion

The data showed that physically active participants have higher FM-adjusted FFM already during growth under age 18 y. Thus, physical activity is a major determinant of body composition as reflected in FFM in this cross-sectional analysis. However, older age counteracted the positive association of physical activity with FFM. Peak FFM was observed around age 30 y, in females and in males (Figure 2).

Age of unadjusted peak FFM is clearly higher than age of peak bone mass, in females at 19–20 y and in males at 20–24 y, independently of race (5). The higher age for unadjusted peak FFM than for peak bone mass is probably explained by FM-associated FFM. FM was highest in females around age 50 y and in males around age 75 y (Figure 2). Thus, FM-associated FFM dominated the decrease in physical activity–associated FFM in participants with a higher FM.

A previous study found the age of unadjusted peak FFM to be in the mid-40s for females and mid-20s for males (13). In the current study, peak FFM was at ∼30 y old for both males and females, a difference possibly explained by differences in FM and thus in FM-associated FFM between the populations of study.

In adults, larger FFM in participants with a larger FM follows the quarter FFM rule (25). On average, 24% of the higher body mass was FFM. Factors confounding the quarter FFM rule, including an extreme imbalance between energy intake and energy expenditure and effects of differences in physical activity, were excluded in the model as presented. All participants were observed under neutral conditions of energy balance, and measured PAL was included in the model as an independent variable. Unfortunately, the quarter FFM rule still lacks a mechanistic explanation (27).

The controversy on FFM maintenance through physical activity at later age seems to be at least partly explained with inclusion of differences in FM between participants, in the model as presented. FFM adjusted for differences in FM was significantly higher in participants with a higher PAL, for females and males at younger age. The mean difference of 3.6 kg (95% CI: 2.8, 4.4 kg) and 4.4 kg (95% CI: 3.2, 5.7 kg) FFM at age 18 y and 0.7 kg (95% CI: −0.2, 1.7 kg) and 1.0 kg (95% CI: −0.1, 2.1) FFM at age 80 y, as calculated from the model presented, between a female and male with PAL = 1.5 and 2.0, respectively, is in line with an earlier cross-sectional analysis. Manini et al. (18) observed (mean ± SD) 2.0 ± 1.2 kg and 2.9 ± 1.3 kg greater FFM in older females and males, respectively, in participants in the first than in those in the third tertile of doubly labeled water–assessed activity energy expenditure. Differences in FM between participants in the first and third tertiles of activity energy expenditure were nonsignificant. However, despite a greater FFM in participants with a higher PAL, the age-related decline in FFM might not be prevented by a higher PAL.

In a 5-y follow-up of the participants 70–82 y old observed by Manini et al. (18), changes in physical activity did not affect the age-related change in body composition.

The average difference between peak FFM and FFM at age 80 y, an age interval where PAL remained the same, was −8 kg (Figure 2). The 8-kg difference between peak-FFM and FFM at age 80 y is similar to an earlier identical cross-sectional comparison resulting in −7.5 kg and −8.8 kg difference for females and males, respectively (30, 31). The mean difference in FM-adjusted FFM between a sedentary and a very active participant over the same age interval was between 3 and 4 kg. At older age, despite a greater routine physical activity, the inverse association of age*PAL counteracts the positive association of PAL with FFM.

Although aerobic exercise does not completely prevent the lower FFM in aging participants, resistance exercise may be more helpful (32). However, although resistance exercise elicited an ∼1-kg increase in FFM among older adults, this is modest compared with the differences with healthy young adults and with the 8-kg difference aforementioned (33). Exercise training in adults at older age has little or no effect on muscle mass but is important for physical fitness and performance (34). Physical activity and exercise training increase functional capacity, allowing individuals to maintain their independence with increasing age and participate in activities associated with daily living (35).

One major cause of muscle mass loss with aging appears to be the alteration in hormonal activity involved in muscle regeneration and protein synthesis (36). Hormone replacement therapy in women is shown to diminish age-associated muscle loss and to raise the synthesis rate in skeletal muscle after exercise training (37). Thus, age-associated hormonal activity is one explanation for the age-associated interaction between physical activity and FFM.

From a longitudinal point of view, physical activity during growth may provide lifelong benefits by reaching higher peak FFM, as shown for physical activity and lifelong bone health (38, 39). Development of FFM and bone mass may be coordinated (40). The growth phase is a window of opportunity for achieving higher peak FFM to maximize bone mass, through a physically active lifestyle (41). If longitudinally confirmed, early-life physical activity may contribute to prevention of disease in old age (42).

The study has several strengths. It was conducted in a large participant group (i.e., 2000 participants) covering early to late life, obtaining physical activity from doubly labeled water–measured energy expenditure and FFM from total body water as measured with isotope dilution, both considered gold-standard methods. An obvious limitation is the observational design. In addition, the use of the 2-compartment model of body composition cannot discern the difference in changes of separate components of FFM, including muscle mass and FM-associated FFM.

In conclusion, physically active participants show higher FM-adjusted FFM, especially after growth at age 18 y. Thus, physical activity seems to be a major determinant of body composition as reflected in peak FFM. Older age counteracts the positive association of physical activity with FFM.

## Data Availability

Data All data used in these analyses are freely available via the International Atomic Energy Agency Doubly Labeled Water database (https://www.dlwdatabase.org/).

## Notes

Supported by US National Science Foundation grant BCS-1824466 (to HP). The funders played no role in the content of this article.

JP-R is deceased.

Abbreviations used: FFM, fat-free mass; FFMI, fat-free mass index; FM, fat mass; FMI, fat mass index; PAL, physical activity level; REE, resting energy expenditure; TEE, total energy expenditure.
